# Early childhood obesity prevention efforts through a life course health development perspective: A scoping review

**DOI:** 10.1371/journal.pone.0209787

**Published:** 2018-12-28

**Authors:** Sheri Volger, Diane Rigassio Radler, Pamela Rothpletz-Puglia

**Affiliations:** Department of Clinical and Preventive Nutrition Sciences, School of Health Professions, Biomedical and Health Sciences, Rutgers University, Newark, New Jersey, United States of America; Teesside University, UNITED KINGDOM

## Abstract

**Introduction:**

The obesity rate in preschool children in the United States (US) is 13.9%, while even higher rates are associated with racial and ethnic minorities and children from low-income families. These prevalence patterns underscore the need to identify effective childhood obesity prevention programs.

**Method:**

A scoping review was conducted following Arksey and O’Malley’s framework to provide an overview of the types, effectiveness and cost-effectiveness of obesity prevention interventions and policies in children up to 6 years old. Inclusion criteria were studies at least 6-months duration; included a weight-based outcome, conducted in the US, English publications from January 2001 to February 2018. Exclusions: studies in overweight/obese children and obesity treatments, no comparator group. Evidence was characterized across the early life course and multiple-levels of influence.

**Results:**

From the 2,180 records identified, 34 met the inclusion criteria. Less than half of the interventions initiated during pregnancy, infancy or preschool reported a significant improvement in a weight-based outcome. All interventions included strategies to influence individual- or interpersonal-level health behaviors, yet few removed obstacles in the healthcare system, physical/built environment, or sociocultural environment. The majority (78%) of the interventions occurred during preschool years, with 63% conducted in early childcare education settings serving low-income families. The health impact of the state-wide and national policies on children under age 6 years remains unclear. There was considerable uncertainty around estimates of the health and economic impacts of obesity prevention interventions and policies.

**Conclusion:**

There is a need to intensify early childhood obesity preventive efforts during critical periods of health development in the US. Future studies should estimate the feasibility, program effectiveness, and cost of implementing multilevel obesity prevention interventions and policies. Addressing these research gaps will provide stakeholders with the scientific evidence necessary to facilitate funding and policy decisions to decrease the prevalence of early childhood obesity.

## Introduction

Despite recommendations to prioritize obesity prevention efforts, [1–4] epidemiological data from the 2015–2016 National Health and Nutrition Examination Survey (NHANES) found that the prevalence of early childhood obesity remains at a 10-year high [5]. Furthermore, obesity rates among school-aged children aged 6–11 years are approximately 25% higher compared with preschool children aged 2–5 years [5]. In addition, even higher obesity rates are differentially associated with minorities and children from low-income families [6]. These prevalence patterns underscore the need to focus on early childhood obesity prevention efforts with the goal of meeting the Healthy People 2020 obesity rate target of 9.4% [2]

**Fig 1:**
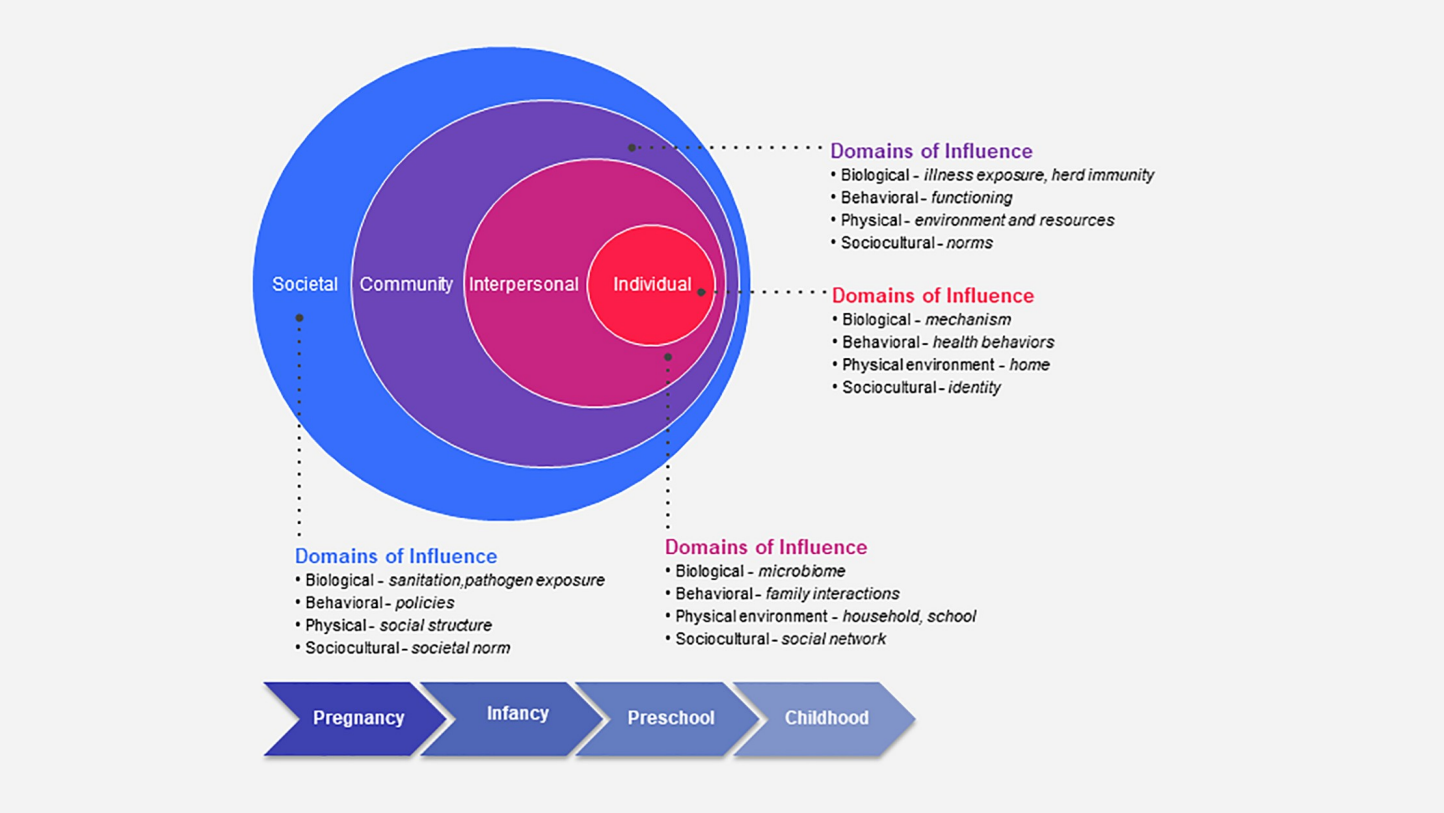
Framework used to characterize components of early childhood obesity prevention interventions across the early life course. Adapted from The National Institute on Minority Health and Health Disparities (NIMHD) Research Framework [9].

The evaluation of such efforts should be guided by framework models that consider the various levels that influence an individual’s health trajectory [7, 8]. For example, the National Institute on Minority Health and Health Disparities (NIMHD) Research Framework represents the multiple levels of modifiable and interacting determinants that contribute to health disparities (Fig 1) [9]. While the multi-level Life Course Health Development Framework perspective also recognizes that health-development unfolds over the life course, is sensitive to time and environment, adaptive, and requires a balance among all levels of health [10]. Together, these models are well-suited for evaluating and characterizing childhood obesity prevention efforts and informing future interventions.

We conducted a scoping review to provide an overview of the current state of obesity prevention efforts in the United States (US) with children less than 6 years of age, and to answer two questions: “What types of interventions and policies are being used for obesity prevention across the early life course and at multiple levels of influence?” and “How effective are they?” The secondary aim was to describe the best available evidence on the cost and cost-effectiveness of implementing obesity prevention interventions and policies.

## Methods

A scoping review was conducted to expand on previous systematic and narrative reviews of obesity prevention efforts in young children and to identify scientific evidence from a broad range of interventional studies, government and non-governmental programs, and local and national policies in the US. The scoping study design was chosen because it offers a framework to identify, “map”, merge evidence, and synthesize a broad range of evidence. Furthermore, the scoping review methodology is focused on providing conceptual clarity and allows researchers to focus on questions with relevance to target populations and locations [11–12].

The scoping review process is based on the Arksey and O’Malley’s 5-stage methodological framework [11]. The 5-stages that served as a roadmap for the present review are: 1. identifying the research question; 2. identifying relevant studies; 3. study selection; 4. charting the data, and 5. collating, summarizing and reporting the results. [11]. While the Joanna Briggs Institute’s Reviewer’s Manual of best research practice guidelines for conducting a systematic scoping review, served as a guide for the present review [12]. To ensure consistency, transparency, and reproducibility an a *priori* scoping review protocol was developed and directed the review process.

### Data sources

After formulating the review objectives and the research questions (Stage 1), a literature search was conducted to identify relevant studies (Stage 2) in the Cochrane Central Register of Controlled Trials, MEDLINE PubMed, CINAHL, PsycINFO, and EconLit databases from January 2001 to February 2018. The timeframe was chosen because in 2001 the Department of Health and Human Services published “The Surgeon General's Call To Action To Prevent and Decrease Overweight and Obesity” [13] prioritizing the public health response to the growing obesity epidemic.

### Search strategy

A search strategy was devised with the assistance of an Information & Education Librarian (MG) for PubMed using keywords from obesity prevention articles and modified for the additional electronic databases. Table 1 shows the search syntax and strategy.

**Table 1:** Search strategy and study selection criteria.

**Search Strategy**		
**PubMed Search Strategy**	((policy[Title/Abstract] OR policies[Title/Abstract] OR prevention[Title/Abstract] OR "primary prevention"[Mesh]) AND ("Child, Preschool"[Mesh] OR "Infant"[Mesh] OR childhood[Ti] OR childcare[Ti] OR early childhood[Ti] OR preschool[Ti]) AND (obesity[mh] OR obese[tiab] OR obesity[tiab] OR overweight[tiab] OR over-weight[tiab])) NOT (("Review"[Publication Type] OR "Meta-Analysis"[Publication Type] OR "Meeting Abstracts"[Publication Type] OR "research design"[Mesh]))	
**Limits**	Full text; Publication date from 2001/01/01; Humans; English; Newborn: birth-1 month; Infant: birth-23 months; Infant: 1–23 months; Preschool Child: 2–5 years	
**Study Selection Criteria (PICOTS)**		
	**Inclusion Criteria**	**Exclusion Criteria**
**P****opulation**	-Children under the age of 6 - Records/ separate analysis of children under the age of 6, if older children were also included -Otherwise healthy	-Populations of exclusively obese or overweight children -Children with a chronic illness, disability, congenital malformation or significant medical condition that could contribute to changes in body weight
**I****nterventions, Policies or Practices**	Any intervention, program or policy aimed at preventing early childhood obesity	-Interventions, programs or policies aimed at obesity treatment or promoting weight loss
**C****omparison**	-Other intervention, programs or policy aimed at preventing early childhood obesity -Or usual care, within the same aged children and in a similar setting -Or an equivalent historical control group or policy period	-No relevant comparator group
**O****utcome**	Change in a weight-based measure of growth or weight status such as weight, weight/weight percentiles, BMI / BMIz-score, or BMI categories (underweight, normal weight, overweight, obese) compared to a comparator group	-No weight-based outcome measure
**T****iming**	-Interventions/programs lasting at least 6 months long or collecting a weight-based outcome measure 6 months after initiation -Policies since 2001	- Interventions/programs less than 6 months
**S****etting**	-Individual (primary-care based) -Interpersonal (home-based, peers) -Community (childcare-based, community organizations) -Societal (policies, health information, social norms)	-Non-US settings or policies
**Year Range**	Publications from Jan 2001 to Feb 2018	-Publication prior to 2001
Studies estimating implementation costs, cost effectiveness analyses and policy studies were included provided that the assumptions and datasets included children under 6 years of age.		

### Study selection

During study selection (Stage 3), publication titles and abstracts were screened, duplicates deleted, and full-text articles reviewed for eligibility based on the review’s inclusion criteria. References from the bibliographies of included trials were hand searched. Two researchers (SV, PRP) independently reviewed, discussed, and agreed upon the eligibility of all studies. While systematic reviews adhere to rigid inclusion criteria, scoping studies’ inclusion criteria are broad to allow for the evaluation of a wide range of information [12]. Eligible studies were included if they incorporated a comparator group; were conducted in children with a normal or healthy weight (BMI-for-age percentile between the 5th percentile to less than the 85th percentile); children under the age of 6 or woman in any setting, and reported at least one weight-based outcome measure of growth or weight status (Table 1). While critical appraisal of methodology is not the focus of scoping reviews, we followed a standardized research protocol and applied the Dixon Woods threshold to exclude articles judged "fatally flawed" [14].

### Data extraction

Data extraction (Stage 4) was done using a two-step process. First, a Microsoft Excel, version 2016 (Microsoft, Redmond, WA) data extraction template was developed to chart continuous and categorical variables and perform summary statistics. S1 Appendix shows a list of the key data extraction variables. Next, included articles were imported into Nvivo 11 Pro (QRS International, Doncaster, Australia) and qualitative data were extracted by selecting, coding and creating nodes (files) representing key concepts. A coding structure and organizational hierarchy was created to characterize major themes by life course, concepts and context pertaining to the NIMHD Framework (Fig 1).

## Results

### Collating, summarizing and reporting the results (Stage 5)

**Fig 2:**
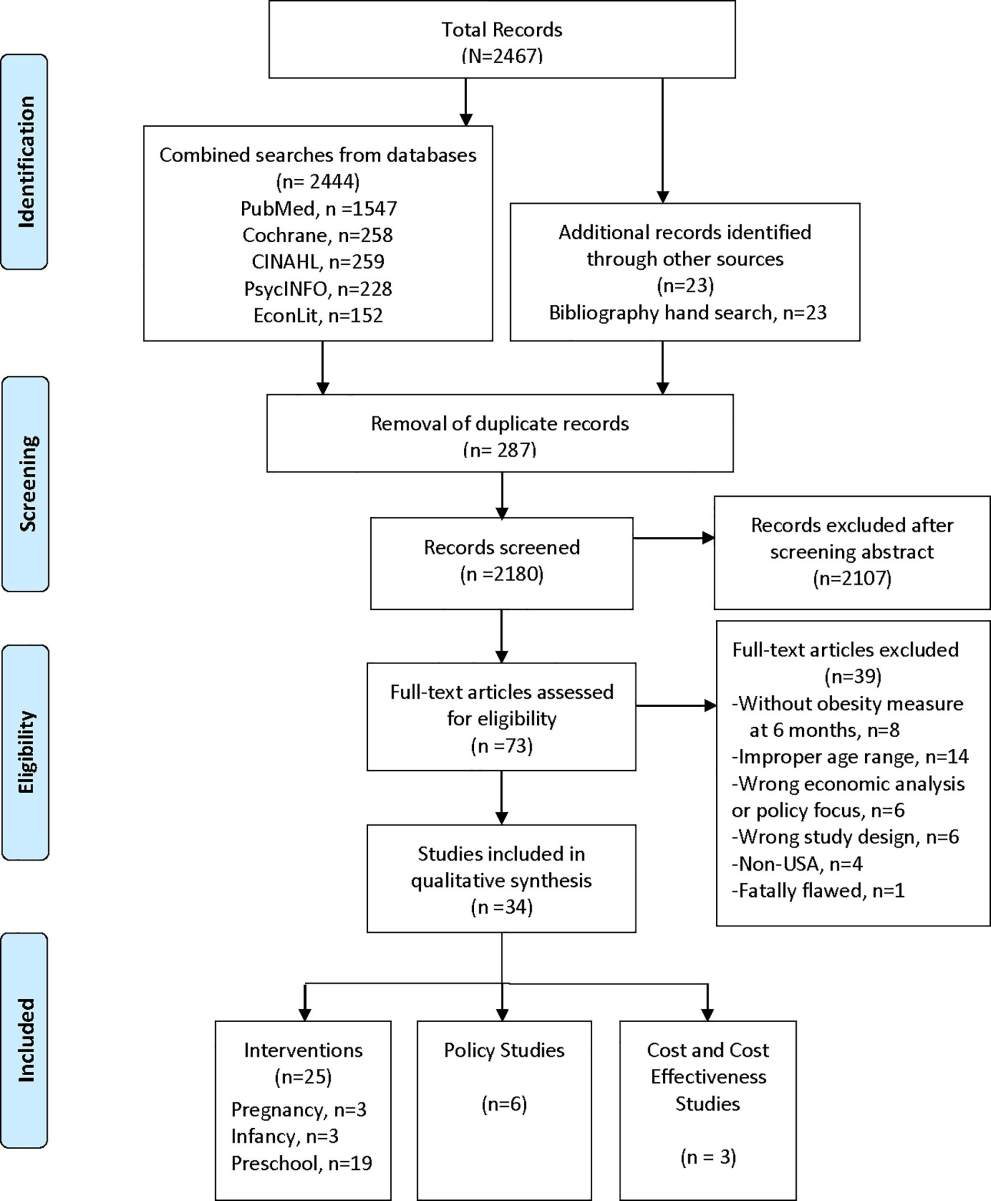
Flow diagram showing literature and study selection. Adapted from Moher D, Liberati A, Tetzlaff J, Altman DG. Preferred reporting items for systematic reviews and meta-analyses: the PRISMA statement. BMJ (Clinical research ed). 2009;339:b2535, [15].

Fig 2 describes the literature search and study selection process. We identified a total of 2,467 records. After removing duplicate records, the titles and abstracts of 2,180 records were screened for inclusion. The full text of 73 articles were reviewed for eligibility and 34 studies were included in the review.

### Study characteristics

The included studies examined the collective experiences of approximately 900 pregnant women, 1,600 infants and 10,000 preschool aged children across 16 states, in 10 urban centers, and a mix of suburban and rural communities (Tables 2–4). The interventions (n = 25) were initiated during three stages in the early life course: pregnancy (n = 3),[16–18], infancy (n = 3), [19–21] and preschool (n = 19), [22–40]. The majority (88%, 22/25) of the interventions used an experimental study design (19–40).

**Table 2:** Characteristics of interventions during pregnancy.

Author Year	Study location	Population	Study Design	Theoretical Framework	Level of Influence	Intervention	Participant	Effectiveness
Study Name	Study Setting		Analytical Sample ^a^		Domains of Influence	Delivery	Treatment provider	
Gregory [16] 2016	Baltimore, MD	Pregnant, < 21 weeks gestation; Pre-pregnancy BMI > 30 kg/m^2^; Medicaid insurance; Mainly AA	Retrospective cohort	Obstetrical model	Individual, interpersonal	Behavior, Diet, Appropriate GWG	Mother, Infant	Not significant: at 1-year, infant WFL ≥ 95th percentile I = 17%; C = 15%, P = .66
Nutrition in Pregnancy clinic (NIP)	Urban hospital, health care clinic		Mothers: I = 61; C = 145 Infants: I = 32 C = 97		Biological, Behavioral, Healthcare system	Individual sessions	Physician, Nurse, practitioner, Nutritionist	
Olson [18] 2014	Rural, NY 8-counties with below state median family income; higher rates of childhood overweight/ obesity in 6 of the counties	Pregnant, <24 weeks gestation and 6 months post-partum; infant weights through 6 months	Prospective cohort study	Community coalition action theory	Individual, Interpersonal, Community, Societal	Environmental community changes, Diet, PA, Appropriate GWG, Breastfeeding	Mother, Infant, Community	Not significant: at 6 months, WFL z-score in (%), I = 34.2%; C = 31.4%, P = .52
Healthy Start Partnership (HSP)	Combination community- wide plus primary care clinic		Mothers: I = 114; C = 152; Infants: I = 88 C = 65		Biological: Behavioral, Physical / built, Sociocultural	Community exposure, multiple formats & modes of delivery	Multiple sectors	
Karanja [17] 2010	Portland Area Indian Health Services (Idaho, Oregon, WA)	Pregnant: affiliated with 1 of 3 AI / AN tribes with children at higher risk for overweight	Before and after design	Home-visiting model	Individual, interpersonal, community	Environmental community changes, Behavior, Diet, Breastfeeding, Reduce SSB	Mother, Infant, Community	**Significant**: at 24 months, BMI- z scores increased less in the community-wide plus home groups (Tribes B & C) compared with community only (Tribe A) (−0.75, P = .016)
The toddler overweight and tooth decay prevention study (TOTS)	Combination community-wide plus home-visit/ phone		Mothers: I = 142; C = 63; Infants: I = 125; C = 53		Biological, Behavioral, Physical/ built, Socio-cultural, Healthcare system	Individual, face to face, Phone, Multimedia	Trained, peer, community worker	

Abbreviations: AA, African American; AI, American Indian; AN Alaskan Native; BMI, Body Mass Index kg /m^2^, C, comparator group; GWG, gestational weigh gain; I, Intervention group; PA, physical activity; PCP, primary care provider; WFL, weight-for-length; Wt, weight

^a^ Sample size is the analytical sample or sample included in the primary analysis

**Table 3:** Characteristics of interventions during infancy.

Author Year	Study location	Population	Study Design	Theoretical Framework	Level of Influence	Intervention	Participant	Effectiveness
Study Name	Study Setting		Analytical Sample ^a^		Domains of Influence	Delivery	Treatment provider	
Machuca [19] 2016	Bronx, NY	Enrolled in WBG by age 2 months; Attended at least on WBG group and 24- or 30-month well-child care visit	nRCT	Trans-theoretical model stages of change; Social Learning Theory	Interpersonal, Individual, Community	Behavior and Diet	Mothers, Infant	**Significant**: at 2 years, I group were significantly less likely to have a BMI-for-age ≥ 85th percentile compared with C (2.1% vs. 15.0%; OR 0.12; 95% C; 0.02–0.94; P = .02)
Well Baby Group (WBG)	Federally Qualified Healthcare Center		Mothers: I = 47; C = 140		Biological, Behavioral, Sociocultural environment, Healthcare system	Group mother sessions	Pediatrician Registered Dietitian	
Schroeder [21] 2015	Baltimore, MD	Healthy infants; ≥2000 g birth weight; discharged home < 5 days post birth	Randomized cluster	Not stated	Individual, Interpersonal	Behavior, Diet and PA	Parents, Infant	Not Significant: at age 24 months, no between group difference in growth pattern; for example, mean (SD) BMIz-scores I = [0.339 (1.13) vs.C = 0.218 (0.95), P >.05]
Growing Leaps and Bounds	Health Centers		Centers = 4; Infants: I = 112; C = 110		Biological, Behavioral, Healthcare system	Individual sessions, Brochures, Phone, Postcards	Pediatrician; Nurse practitioner; Clinic staff	
Paul [20] 2011	Hershey, PA	Mothers intending to breastfeed; Newborn infants	RCT (2 x 2 design)	Not stated	Interpersonal, Individual	Behavior and Diet	Mothers, Infant	**Significant**: at 1 year, infants in both I groups had lower mean WFL percentiles compared with C (I = 33^rd^ percentile vs. C = 50^th^ percentile; P = .009)
Sleeping and Intake Methods Taught to Infants and Mothers Early in life (SLIMTIME)	Home		Mother Infant pairs = 110; Soothe/Sleep, n = 29; Introduction of solids, n = 29; Both, n = 22; None, n = 30		Biological, Behavioral	Individual sessions, face-to-face, video, Instructional handouts	Research Nurse	

Abbreviations: BMI, Body Mass Index kg /m^2^, C, comparator group; I, Intervention group; nRCT, non-randomized control trial; OR, Odds ratio; PA, physical activity; RCT, randomized control trial; SD, Standard deviation; WFL, weight-for-length; WIC, Women, Infants, and Children Program; Wt, weight

^a^ Sample size is the analytical sample or sample included in the primary analysis

**Table 4:** Characteristics of interventions during preschool.

Author Year	Study location	Population	Study Design	Theoretical Framework	Level of Influence	Intervention	Participant	Effectiveness
Study Name	Study Setting		Analytical Sample ^a^		Domains of Influence	Delivery	Treatment provider	
Alkon [22] 2014	California, Connecticut, North Carolina	3–5 years, mainly low income	Cluster RCT (efficacy)	Not Stated	Individual, Interpersonal	Behavior, Diet, PA, and Policy	Child care providers; Other staff; Parents	**Significant:** at 7 months, significant difference in child-level change in mean BMIz-scores between I and C: Multilevel mode (HLM)l: coeff(SE) -0.14 (0.06); [95% CI; (−0.26, -0.02)]; t statistic (df)(-2.54); P = .02
Nutrition and Physical Activity Self-Assessment for Child Care	Licensed child care centers:		Licensed child care centers: I = 9; C = 8; Children: I = 99; C = 110		Biological, Behavioral, Physical/ Built	Group child sessions; Parent information sheets; Policy changes and consultation sessions	Trained nurse; Child care health consultants	
Annesi [23] 2013	Southeast USA/ Atlanta, Georgia	4–5 years; in final year of YMCA affiliated preschool; AA; income at or below US 130% poverty line	Cluster RCT (Efficacy)	Social cognitive; Self-efficacy theory	Individual, Interpersonal	Behavior and **PA**	Child; Parental support	**Significant**, at 9 months mixed model repeated-measures ANOVA found significant time x treatment effect [F (df 1, 271) = 4.49, P = 0.035) indicating a greater reduction in the change in BMI for the I group
Start for Life	Childcare center		YMCA-affiliated preschools: I = 9; C = 8 Children: I = 144; C = 129		Biological, Behavioral	Group child exercise, Activity logs, Certificate of accomplishment	Childcare teacher trained	
Annesi [24] 2013	Southeast USA	4–5 years, in final preschool year; mainly AA (86%); lower to lower-middle class	Cluster RCT (Efficacy)	Social cognitive; Self-efficacy theory	Individual, Interpersonal	Behavior and **PA**	Child; Parental support;	**Significant**: at 9 months mixed model repeated-measures ANOVA found significant time x treatment effect [F (df 1,1152) = 5.16, P = 0.023) indicating a greater reduction in the change in BMI for I group
Start for Life	Childcare center		YMCA-affiliated preschools: I = 18; C = 8 Children: I = 690; C = 464		Biological, Behavioral	Group child exercise, Activity logs, Certificate of accomplishment	Childcare teacher trained	
Fitzgibbon [28] 2005	Chicago, Illinois	3–5 years, attends HS; low-income; primarily black (99%)	Cluster RCT (Efficacy)	Social cognitive theory; Self-determina-tion theory; Trans theoretical model-stages of change -	Individual, Interpersonal	Behavior, Diet and **PA**	Child, Parents	**Significant**: a smaller increase in BMI for I vs. C at 1 year [-0.62 (95% CI -0.95, -028), P = .002] and 2 years [-0.65 (95% CI; -1.09 to -0.21) P = .008]
Hip-Hop to Health Jr.	Childcare center		HS Centers: I = 6; C = 6 Children: I = 143; C = 146		Biological, Behavioral, Sociocultural	Group child sessions; Newsletter, Homework	Trained childhood educators (research team member)	
Natale [36] 2017	Miami-Dade County, Florida	2–5 years; low-income children; racial/ ethnic distribution of Miami-Dade County: 60% Hispanic, 20% AA	Cluster RCT (Effectiveness)	Socio-ecological model; Social Cognitive Theory	Individual, Interpersonal	Behavior, Diet, PA and Policy	Child care providers and staff; Parents, Child	**Significant:** at 2 years, a growth curve analysis showed a significantly smaller increase in PBMI for I vs. C [negative slope (ß coefficient = -1.95, SE = 0.97, P = .04)
Healthy Caregivers-Healthy Children	Childcare center		Subsidized Child care centers: I = 12: C = 16: Children: I = 754; C = 457		Biological, Behavioral, Physical/ Built, Sociocultural	Policy changes; Group teacher, parent and child sessions; English and Spanish resources; Newsletters, Homework,	Childcare teacher trained; Bilingual study team members;	
Lumeng [34] 2017	Urban and Rural Michigan	Child attending HS, first year	Cluster RCT (Efficacy)	Social cognitive theory; observational learning/ reinforcement techniques	Individual, Interpersonal,	Behavior and Diet	Child; Parents	Not significant: at the end of the academic year, no between group difference in the prevalence of overweight or obesity and BMI-z scores (All, P >.05)
Preschool Obesity Prevention Series [POPS]	Childcare center		HS classes: I = 9; C = 9; I Obesity-prevention, n = 221; I2 = plus self -regulation, n = 253); C = 216		Biological, Behavioral, Sociocultural	Group child and parent sessions, Video vignettes, Homework, Phone calls	Master’s-level nutrition/ mental health specialist; Childcare teacher trained	
Kong [33] 2016	Chicago, Illinois	3–5 years, HS serving AA, low-income families	Cluster RCT (Effectiveness)	Social cognitive theory; Self-determination theory; Trans-theoretical model -stages of change	Individual, Interpersonal	Behavior, Diet and **PA**	Child, Parents	Not significant: at 1 year, no between group difference in adjusted mean changes in BMIz scores (P = .83)
Hip-Hop to Health Jr.	Childcare center		HS centers: N = 18; Children; I = 285; C = 258		Biological, Behavioral, Sociocultural	Group child sessions: Exercise CD, Newsletter, Homework	Childcare teacher trained	
Esquivel [27] 2016	Oahu, Hawaii	HS classroom; 2 to 5 years; NHPI children (23%)	Cluster RCT (Effectiveness)	Not stated	Individual, Interpersonal,	Behavior, Diet, PA and Policy	HS Teachers; Child	Not significant: at 7 months, no within -group differences in mean change in BMIz-scores and BMI categories (All, P >.05)
Children’s Healthy Living Program (CHL)	Childcare center		HS classes (geographical cluster): I = 11; C = 12; Children: I = 114; C = 132		Biological, Behavioral, Physical/ Built	Group teacher sessions, Menu changes, Classroom nutrition / PA resources, Newsletters, Phone	Childcare teacher trained	
Natale [35] 2014	Miami-Dade County, Florida	2 to 5 years, child care centers serving multi-ethnic children from low-income families	Cluster RCT (Effectiveness)	Socio-ecological model	Individual, Interpersonal	Behavior, Diet, **PA** and Policy	Child care providers; Other staff; Parents; Child	Not significant: at 12 months no between group difference in mean Wt. (P = .35) and BMI-z scores (P = .81)
Healthy Inside–Healthy Outside	Childcare center		Subsidized child care centers: I = 6; C = 2; Children: I = 238; C = 69		Biological, Behavioral, Physical/ Built, Sociocultural	Policy changes; Group sessions, Spanish and English resources; Newsletters, Homework	Childcare teacher trained; RD/ Nutritionist	
Fitzgibbon [30] 2013	Chicago, Illinois	3–5 years, low-income, Latino	Cluster RCT (Feasibility)	Social cognitive theory; Health belief model; Self-Determination Theory	Individual, Interpersonal	Behavior, Diet and **PA**	Child, Parents	Not significant: at 1 year, did find a greater reduction in in BMI and BMIz- scores in the I group (P >.05)
Family-based Hip-Hop to Health	Childcare center		HS centers: I = 2, C = 2: Children: I = 61; C = 67		Biological, Behavioral, Sociocultural	Group child sessions, Nutrition and Spanish exercise CD, Parent group sessions and PA, Newsletters	Trained, bilingual/ bicultural educator	
Fitzgibbon [29] 2006	Chicago, Illinois	3–5 years, low income; mainly Latino HS centers	Cluster RCT (Efficacy)	Social cognitive theory; Self-determination theory	Individual, Interpersonal	Behavior, Diet and **PA**	Child, Parents	Not significant: at 1 and 2 years, no between group differences in change in BMI and BMIz-scores (P = .05)
Hip-Hop to Health Jr. Latino	Childcare center		HS centers: I = 6, C = 6; Children: I = 176; C = 160		Biological, Behavioral, Sociocultural	Group child sessions; Lessons in English and Spanish: Spanish exercise CD; Newsletter, Homework	Trained, bilingual/ bicultural early childhood educator (research team member)	
Haines [32] 2016	Boston, MA	2–5 years; Hispanic (58%) and Black/AA (23%) recruited from community resources serving low-income families	RCT (Efficacy)	Social contextual framework	Individual, Interpersonal	Behavior, Diet and **PA**	Parents, Child	Not significant: at 9 months, no between group difference in BMI (P = .41)
Parents and Tots Together	Community health center		Parent-child dyad: I = 46, C = 50		Biological, Behavioral	Group child and parent sessions, DVD set, Newsletter, Homework, (bilingual interviews)	Trained facilitator	
Slusser [38] 2012	Los Angeles, CA	Parent of 2–4 years; Low-income Latino	RCT (Pilot)	Social learning framework	Individual, Interpersonal	Behavior, Diet and PA	Mother, Child (Wt. only)	**Significant:** at 1 year, decrease in mean (SD) PBMI in the I group [-3.85 (0.29) vs. an increase in C group [+1.33 (0.30)]; accounting for drop-out rates with multiple imputation, significant difference shown between change in BMI-z scores [0.24 (0.01) P < .04]
Pediatric Overweight Prevention through Parent Training Program	Community center/ health centers		Local centers: Parent-child dyad: I = 44; C = 37		Biological, Behavioral, Sociocultural	Group, Spanish, parent training sessions; Spanish handouts; Homework	Trained, bilingual staff; social worker or master’s level health educator	
Cloutier [25] 2015	Hartford, CT	Caregiver of 2–4 years; Hispanic (82%) / AA WIC recipient	N-RCT (Efficacy)	Chronic care model	Individual, Interpersonal	Behavior, Diet and PA	Mother, Child (Wt only)	**Significant:** at 12 Months, significant intervention effect on change in PBMI (ß coefficient = -0.23; 95% CI; -0.33, -0.13) with a mean decrease in I (-0.33) compared with an mean increase PBMI (8.75) in the C group (P < .001)
Steps to Growing Up Healthy	Pediatric Primary Care Clinic		Clinics, N = 32; Parent-child dyad: I = 200, C = 218		Biological, Behavioral, Sociocultural Healthcare System	Individual MI sessions, English and Spanish resources; Handouts, Self-monitoring calendar, Toolkit	Trained primary care clinicians and nurses; bilingual team members	
Sherwood [37] 2015	Minneapolis-St Paul area	Families with a 2- to 4-year-old with a scheduled well-child visit; BMI or weight-for-height age and sex percentile from 50th to 95^th^; one overweight parent	RCT (Pilot)	Social ecological models; Social cognitive theory	Individual, Interpersonal	Behavior, Diet and PA	Parent; Child (Wt. only)	Not Significant: at 6 months no difference in PBMI (P = 0.64) and BMI z-scores (P = 0.89); post hoc analysis of baseline child weight status moderated the time by treatment effect on BMI percentile (P = .04)
Healthy Homes/ Healthy Kids- Preschool	Pediatric Primary Care Clinics/ Phone		Parent-child dyad I = 30, C = 30		Biological, Behavioral, Healthcare system	Individual MI session, Flipchart, Handouts: Phone MI sessions	Pediatric PCP counseling; clinic staff; Trained coaches	
Woo Baidal [40] 2017	Fitchburg and New Bedford, MA	2–4 years, WIC participant	N-RCT (Efficacy)	Chronic care model; Energy gap model; Social cognitive theory	Individual, Interpersonal, Community	Behavior, Diet and PA	WIC -Providers; Parents Child (Wt. only)	Not Significant: Over 2 years, no significant difference in BMIz-scores adjusting for age, gender, race, ethnicity (P>0.05); Sensitivity analysis, excluding Asian children found site I2 had a significant decrease in BMI-z scores [-0.08 units/year (95% CI, -0.14, -0.02), P = 0.01] compare with the C group
MA-CORD WIC	WIC Sites		WIC centers I = 2, C = 1 Children: I site1 = 198; I site2 = 637; C = 626		Biological, Behavioral Healthcare system	Train-the-trainer group sessions, Individual parent sessions, Handouts; Healthy weight clinic referrals	Trained WIC providers	
Davis [26] 2016	Albuquerque NM	Under 4-years followed for 1 to 2 years; HS serving rural, Hispanic and AI, low-income families	Cluster RCT (Efficacy)	Social ecological model	Individual, Interpersonal, Community	Behavior, Diet, **PA** and Policy	Child; Parents; Family; HS teachers and food service	Not significant: at 6 months, no between group difference in change in mean BMIz-scores (P = .69) and 2 years (P >.30)
Child Health Initiative for Lifelong Eating and Exercise (CHILE)	Childcare center plus local community component		HS centers: N = 16; Children I = 945, C = 871		Biological, Behavioral Physical/ built, Sociocultural, Healthcare system	Group child sessions, English and Spanish resources; Teacher and foodservice training; Family events; Grocery store component, Healthcare provider support	Childcare teacher trained; Grocery store; Healthcare provider	
Haines [31] 2013	Boston, MA	2–5 years; low-income; racial / ethnic minority; television in the child’s bedroom	RCT (Effectiveness)	Not stated- applied (MI coaching)	Individual, Interpersonal	Behavior and Diet	Families, Child	**Significant**: at 6 months, mean BMI decreased in I group (-0.18) but increased in C group (+0.21) with a difference of −0.40 (95% CI, −0.79 to 0.00; P = .05).
Healthy Habits, Happy Homes	Home/ phone		Parent-child dyad: I = 55; C = 56		Biological, Behavioral, Physical/ built, Sociocultural	Individual, home MI sessions, Phone, Mail, Text messages	Trained, bilingual, health educators	
Sun [39] 2017	San Francisco Bay Area, CA	Child: 3–5 years; attends HS; Low income, Chinese mothers speak / read Cantonese with a BMI ≥ 23 or waist circum. >31.5	RCT (Pilot)	Information Motivation behavior model	Individual, Interpersonal	Behavior, Diet and PA	Mothers, Child (Wt. only)	Not Significant: at 6 months, no difference in post-baseline assessment in child’s BMI (t = 1.21, P = 0.24)
No study name provided	Internet-based		Parent-child dyad: I- = 16; C = 16		Biological, Behavioral, Sociocultural	Online/tablets computer: Interactive, Cantonese, modules, Animated short videos, Talk show format	Lessons developed by bilingual/ bicultural RDs and health educators	

Abbreviations: AA, African American; AI, American Indian; BMI, Body Mass Index kg /m^2^, C, comparator group; CI confidence interval; coeff(SE), coefficient estimate (standard error); circum, circumference; f, f-test statistic; GWG, gestational weigh gain; HLM, Hierarchical linear modeling; HS, Head Start; I, Intervention group; MI, Motivational Interview; NHPI, Native Hawaiian and Pacific Islander; PA, physical activity; PBMI, BMI percentile; PCP, primary care provider; RCT, randomized control trial; RD, registered dietitian; SD, Standard deviation; t, t-test statistic; vs, versus; WFL, weight-for-length; WIC, Women, Infants, and Children Program; Wt, weight

^a^ Sample size is the analytical sample or sample included in the primary analysis

**Bold PA**- Direct provision of structured PA

We identified 6 publications examining the impact of city, state and national obesity prevention policies [41–46]. Three additional studies calculated the net cost or cost-effectiveness of obesity prevention interventions [47–49].

In total, 11 (44%) interventions reported a positive benefit on a weight-based measure of growth or weight status (e.g., weight, weight/weight percentiles, BMI/ BMI z-score, and BMI categories) [17, 19, 20, 22–25, 28, 31, 36, 38]. The effectiveness of the interventions was inconsistent and contradictory across all stages of the early life course. Tables 2–4 provide descriptions of the characteristics and outcomes of the interventions.

### Studies initiated during pregnancy

This review identified 3 studies initiated during pregnancy conducted in children from low-income families and members of racial-ethnic minority groups who are at higher risk of obesity [6] and in a variety of settings [urban healthcare clinic-bases [16]; community-wide plus home-visits [17]; community-wide plus primary care practice], (Table 2), [18)]. All three interventions focused on similar individual- and interpersonal-level behaviors (preventing excess gestational weight gain [16, 18] and accelerated infant growth [16–18]), two studies also focused on community-level influences [17–18] but only the one study [17] that implemented interventional components at multilevel domains of influence demonstrated a positive effect on Body Mass Index z-score (BMI-z) in American Indian/Alaskan Native tribal communities in the multi-level, community wide-plus home-visit intervention.

### Interventions during infancy

Two [19, 20] of the three [19–21] studies identified were initiated during infancy and showed a positive effect on infant growth (Table 3). All studies included behavioral strategies at the individual and interpersonal level aimed at increasing knowledge of healthy food choices and appropriate growth patterns. The Well Baby Group (WBG) intervention also targeted community levels of influence by delivering sociocultural adapted nutrition education and providing a peer social support network while maximizing the physical environment and healthcare system resources at a federally qualified community health center [19]. At two years, the infants of low-income, predominately Hispanic mothers attending the WBG were significantly less likely to have a BMI-for-age ≥ 85th percentile compared with a randomly selected comparisons group of infants. Also, at one year, infants of mothers who received both the home-based intervention Soothe/Sleep and Introduction to Solids interventions had lower mean weight-for-length percentiles (33^rd^ vs. 50^th^) compared to the no intervention group [20].

### Interventions in preschool aged children

#### Childcare center-based interventions

Over half (63%, 12/19) of the preschool-aged interventions enrolled children from low-income, racially and ethnically diverse families from Head Start centers (n = 7), YMCA-affiliated childcare centers (n = 2) or other subsidized childcare programs (n = 3), (Table 4). The childcare center-based interventions included in this review were designed with interventional components that primarily focused on influencing individual- or interpersonal-level health behaviors of the children and preschool teachers. Only 42% (5/12) of these interventions reported a significant improvement in either BMI-z score [22], BMI [23–24, 28] and BMI percentile [36].

Of the interventions demonstrating a positive effect on BMI, two studies administered 30 minutes of moderate to vigorous physical activity (MVPA) for primarily African American (AA) children attending YMCA-affiliated preschools [23, 24]. Similarly, the “Hip-Hop to Health” efficacy trial [28] enrolled predominately AA preschoolers attending Head Start and used trained educators to deliver 20-minute healthy-lifestyle behavior themed lessons and 20 minutes of directed physical activity (PA). However, the same intervention did not have a beneficial effect on BMI in Latino preschoolers [29], nor did other similar effectiveness trial in Latino [30] and AA preschoolers [33, 50]. Another study found that five, 1-hour long healthy-lifestyle themed workshops presented by trained nurse childcare health consultant to parents, childcare teachers and staff, significantly decreased mean BMI-z scores in children from underserved minority families [22]. Finally, a multilevel, childcare-based intervention [36] showed a significantly smaller increase in the BMI percentile when intervention centers implemented early childcare center policies focused on modifying individual-level child, parent, and teacher behaviors with physical/built and sociocultural environment changes.

#### Primary care providers clinic-based

Two primary care clinic-based studies [25, 37] reported contradictory results. Both studies targeted individual- and interpersonal-level behavioral changes, implemented in a healthcare environment. Only Cloutier and colleagues found a significantly greater reduction in BMI percentile in the intervention group that participated in bilingual, culturally adapted, motivational interviewing (MI) sessions during primary care provider (PCP) visits and phone-coaching session [25].

#### Community center-based

Similarly, two interpersonal-level, family-based studies were conducted a community center-based environment with mixed results. Slusser and colleagues [38] randomized Latino mothers of preschoolers to receive nine culturally tailored, Spanish language, parent-training sessions or be in a Wait List Group (WLG). Despite reporting 33.3% attrition, the intervention group experienced a greater reduction in BMI percentile differences compared with the WLG at nine months. In contrast, Haines and colleagues [32] failed to demonstrate a significant improvement in BMI with a family-based, community health center intervention.

#### Other settings

Additional preschool-aged interventions were conducted at a WIC site [40], online [39] and in the home and over the telephone [31]. Only one study reported a positive effect on BMI. In the “Healthy Habit, Healthy Home” [31] health educators used MI coaching techniques during home and phone coaching sessions, along with text messages to promote interpersonal-level changes in healthy family routines and PA, encourage family meals and beverage choices, adequate sleep and change the physical-built environment by asking families to remove TVs from the child’s bedroom. Although a WIC-based intervention within the community-wide Massachusetts Childhood Obesity Research Demonstration [51] found no effect on BMI-z scores, a post-hoc analysis excluding Asians (due to disproportionate distribution of Asian children in the comparison community) found a significant improvement in BMI-z scores [40].

#### Costs of obesity prevention interventions

Table 5 includes a summary of three studies appraising the cost of preventing obesity. Cradock and colleagues [47] estimated the total annual cost per child associated with implementing and nationally disseminating the PA component of the childcare center-based “Hip-Hop Jr.” PA intervention was $22.65 yearly [33]. Also, Wright and colleagues [45] calculated the net cost of a primary-care based intervention [52], aimed at reducing obesity related behaviors and BMI in overweight and young, obese children at $196 per child [49]. In the third study, Ma and Frick [48] modeled the breakeven point of a hypothetical intervention producing a 1% reduction in the prevalence of obesity among children 0–6 years. Accounting for future medical costs, population-based interventions could cost up to $339 per child and still have a favorable health benefit/cost profile.

**Table 5:** Cost and cost effectiveness of interventions and policies.

Author Year	Study Objective	Setting / Sample	Data source/ Population Reach	Analysis model; Year of Costing^a^	Key evaluation components	Domains of influence	Outcome measures	Net Results: Impact on Obesity and Cost
Evaluation type / Intervention		Location		Discount Rate^a^	Perspective^a^	Levels of Influence		
Ma [48] 2011	Estimate lifetime obesity-related medical costs and establish the breakeven cost saving of obesity prevention intervention	US population	Obesity prevalence estimates from 30 000 000 children ages: 0 to 6 Years; 7 to 12 years; 13 to 18 years; NHANES, 2003–2006; MEPS 2006	Simulation; Year of costing: 2006 US$	Medical cost perspective	Biological, Behavioral, Healthcare system	Preventing and reducing childhood obesity (defined as ≥ 95th percentile of age- and gender BM)	In healthy 0-6-year-old children, spending up to $339 per child will result in a positive cost benefit.
Simulation of an obesity prevention intervention				Discount: medical costs 3% annually		Individual, Interpersonal, Community, Societal		An intervention that results in 1% reduction in obesity in children 0–6 years would result in a $1.7 billion-dollar cost savings
Wright [49] 2014	Estimate the cost of a cluster RCT, obesity prevention intervention to reduce TV viewing time; fast food SSB intake	Non-profit pediatric offices; Eastern, MA	Children 2.0 to 6.9 years old; BMI ≥ 95th percentile or ≥ 85^th^ < 95^th^ percentile with 1 overweight parent (BMI ≥25)	Cost Study: net cost analysis: difference in cost for the I vs. C group); Year of costing: 2011 US$	Costs include: Parent time and costs; Provider’s direct visit-related -costs: 4 chronic care visits; 2 phone calls; Educational materials; Interactive website	Biological, Behavioral, Healthcare system	At 1 year, no significant difference in BMI, kg/m^2^ and BMI z-score; Total I group cost = $65,643 (95% CI, $64,522, $66,842); Total C group cost = $12,192 (95% CI, $11,393, $13,174)	The intervention costs per child, mean I group = $259 (95% CI, $255, $264); C group = $63 (95% CI, $59, $69)
Cost Study: based on the High Five for Kids intervention [51]			I group: Sites (n = 5) Children, (n = 253); C group: Sites (n = 5); Children (n = 192)	Discount: medical equipment 3.5%	Societal perspective	Individual, interpersonal		Net difference in cost between I and C: $196; (95% CI, $191, $202) per child
Cradock [47] 2017	Estimate the cost of a national policy to implement the Hip-Hop Jr. physical activity intervention in licensed childcare centers	Child Care Setting; US population	National Association for Regulatory Administration 2013, Census Bureau, MEPS 2001–2003; Implementation cost estimates from similar intervention	Microsimulation modeling of outcomes and costs; Year of costing: 2014 US$	All intervention costs; State level: training, labor and travel; Program level: training, labor and materials	Biological, Behavioral, Healthcare system	Assumptions based on Hip-Hop results: Reduction in mean BMI (-0.13. SE = 0.11); PA increase in mean mins per day 7.4 (SE = 3.09)	Cases of obesity prevented (2015–2025) 93,065^c^ (95% UI; -88,279, 248,174)
CEA: based on the Hip-Hop to Health Jr. intervention (Kong 2016)		1st year reach: children 3–5 attending licensed child care centers (4.8 million)		Discount: future cost 3% annually	Modified Societal perspective	Individual, Interpersonal, Community, Societal		Cost per BMI unit changed per person $361^c^ (95% UI, $2031, $3454)
Kuo, [41] 2009	Assess the impact of menu-labeling law on population weight gain	Large restaurant chains in LA County, California	LA county Health Survey; California Department of Education Physical Fitness Testing Program (1999 and 2006.) National Restaurant Association	Simulation model	Estimates of total annual revenue, market share, and average meal price of large chain restaurants, total annual revenue;	Biological, Behavioral, Physical/built, Sociocultural	Assumed 10% of customers would order reduced-calorie meals with an average 100 calories reduction	Intervention prevents a total average annual weight gain of 507,500 lbs. in children 5–17 years
Policy, city & county wide law: menu labeling				Health impact assessment approach; weight gain averted	Assumed similar weight gain patterns for all school-aged children aged 5 to 17 years	Individual, Interpersonal, Societal	Estimated annual weight gain in children 5–17 years is 1.25 million lbs.	No cost data
Dharmasena [43] 2012	Estimate the impact of a 20% SSB tax, considering the expected effect on other beverages	Four regions in the US (East, Midwest, South and West).	Nielsen Homescan Panel 1998–2003	Quadratic Almost Ideal Demand System (QUAIDS) model	Estimating direct and indirect effects of a tax on SSB consumption, caloric intake and per capita annual body weight;	Biological, Behavioral	Percent change in per capita consumption of: Regular soda (-49%); High-fat milk (- 2%); Low-fat milk (+ 11%); Fruit Juice (+ 29%); Bottled water (-5%)	Change in body weight, mean -1.54 lbs. per year
					Direct own-price and indirect cross-price effects on other beverages (milk, fruit juice, sports drinks)			
Policy, National: a tax on SSBs						Individual, Interpersonal, Societal	Net calorie reduction: 449.6 calories per person per month.	No cost data
Wright [45] 2015	Estimate the health and economic costs of early childcare center obesity prevention policies	Licensed child care facilities in the US; Eligible population- 6.5 million preschool children	U.S. 2012: 2007 census; Child Care Licensing Study; 2005 NAP; NHANES 2009–2012; US Bureau of Labor Statistics 2013; Agriculture Marketing Service, USDA; Beverage, PA and screen time data from research studies;	Simulation: Markov-based cohort model^b^; Estimated: licensing, training, and beverage costs; Assumed 73% policy adoption rate; Year of costing: 2014 US$	Hypothetical policy intervention: for preschoolers attending childcare centers: Replacing SSBs with water, limiting fruit juice to 6 ounces /child/day, serving reduced fat milk; 90 minutes of MVPA /day; limit screen time to 30 min./week	Biological, Behavioral, Physical/built	Policy components’ contribution to change in BMI: PA (28%); Beverage (32%); Screen time (40%); Short term outcomes: First-year intervention cost ($ million): 4.82 (6.02, 12.6); Ten-year (2015–2025) invention cost ($ million): 8.39 (–10.4, 21.9); Net healthcare cost savings ($ million): 51.6 (14.2, 134)	Total BMI units reduced 338,00 (107,000, 790,000); Mean BMI reduction per eligible preschool child: 0.0186 fewer BMI units (0.00592, 0.0434)
Policy, National: A multi-component early childhood care center policy intervention			Population reach: 6.50 million preschoolers attending childcare	Discount: healthcare costs 3% annually	Societal perspective	Individual, Interpersonal, Societal		ICER, $57.80 per BMI unit avoided; The intervention is 94.7% likely to yield a cost saving by 2025.
Sonnenville [42] 2015	Estimate the impact of eliminating the TV advertising tax subsidy on BMI	US children and adolescents aged 2–19 years	The Nielsen Company; National Longitudinal Survey of Youth; Rudd Report; US Bureau of Labor Statistics 2013 salary; TV viewing/ advertising data from published studies	Simulation: Markov-based cohort model^b^^;^ Year of costing: 2014 US$	CEA of the elimination of the tax subsidy of TV advertising costs for nutritionally poor foods and beverages during children’s programming (> 35% child -audience share)	Biological, Behavioral	Short term outcomes: First-year intervention cost ($ million): 1.05 (0.69, 1.42); Ten-year (2015–2025): Healthcare cost savings ($ millions) - 352 (-581, -138; Net cost saving per dollar spent ($ million): 38.0 (14.3, 74.3)	Total population BMI units reduced among youth 2–19 years (millions): 2.13 (0.83, 3.52); Mean BMI reduction per youth: 0.028 (0.011, 0.046); Estimated reduction in obesity prevalence: 0.30%.
Policy, National: Eliminating the tax subsidy for TV advertising			Population reach: 74 million	Discount: healthcare costs 3% annually	Societal perspective	Individual, Interpersonal, Societal		Two-year costs per BMI unit reduced ($ million): 1.16 (0.51, 2.63)
Long [44] 2015	To quantify health and economic benefits of a national sugar-sweetened beverage excise tax	US population ages 2- adult	NHANES; U.S. Bureau of Labor Statistics 2013; MEPS; Washington and West Virginia State Department of Revenue; SSB intake data from published research studies;	Simulation: Markov-based cohort model^b^^;^ Year of costing: 2014 US$	CEA of the implementing a $0.01/ounce SSB excise tax estimating; The cost and impact of the change in BMI on healthcare costs; Life-years lost DALYs averted; QALYs gained; For the simulation the tax did not apply to 100% juice, milk products, or artificially sweetened beverages	Biological, Behavioral	A tax of $0.01/ounce of SSBs was estimated to result on a 20% (11%, 43%) reduction in baseline SSB consumption; First-year intervention cost ($ million): 51.0 (35.4, 65.5); Ten—year intervention cost (2015–2025). ($ million): 430 (307, 552)-Tax would result in a total healthcare cost savings ($ millions) -23.6 (-54.9, -9.33)	Mean per capita BMI unit reduction for youth 2–19 years of age 0.16 (0.06, 0.37); Estimated 1.38% reduction in youth obesity prevalence rate
Policy, National: SSB Excise Tax			Population reach: 313 million	Discount: healthcare costs 3% annually	Societal perspective	Individual, Interpersonal, Societal		Two-year costs per BMI unit reduced among youth ($ million): 8.54 (3.33, 24.2); Every dollar spent on the intervention would result in $55.0 ($21.0, $140.0) in healthcare cost savings
Toussaint [46] 2017	Examine the impact of the school-based changes, on BMI trajectory in elementary school-aged children over 6 years	6 rural county regions in the Northeast Iowa initiative	Longitudinal cohort data from 4,101 elementary school-aged children (ages 4–12 years)	Linear growth models to determine growth rates; sensitivity analysis to identify program exposure needed to impact BMI growth rates	School policies supporting healthy living, healthy diet and active play; Community resources for healthy, affordable foods; Environment changes to support physical activity and play	Biological, Behavioral, Physical/built	Reported a 0.32 unit increase in BMI (P < .001) for each school grade advanced	Program exposure slowed overall BMI growth rates (P < .05); Program exposure of 1 year or less = BMI growth rate 1.02 (about 5 BMI increase between kindergarten to fifth grade);
Program, regional Northeast Iowa Food and Fitness Initiative			Population Reach: 100,000			Individual, Interpersonal, Community		Program exposure of 2 to 6 years = BMI growth rate of 0.67 (about 3.4 BMI increase from kindergarten to 5th grade); No cost data

Abbreviations: BMI, Body mass index (kg/m^2^); C, Comparator group; CEA, cost-effectiveness analysis; DALYs, disability adjusted life-years; I, Intervention; ICER incremental cost-effectiveness ratio; LA, Los Angeles; MA, Massachusetts; MEPS, Medical Expenditure Panel Survey; NAP, Nutrition and Physical Activity Self-Assessment for Child Care; NHANES, National Health and Nutrition Examination Survey; PA, Physical activity; RCT, randomized control study; SSBs, sugar-sweeten beverages; TV, television; USDA, United States Department of Agriculture; QALYS, quality adjusted life-years.

^a^ Year of costing, discount rate and perspective or other key considerations are shown, if applicable

^b^ Applied modified Australian Assessing Cost Effectiveness (ACE) methodologies using U.S. data, and recommendations from the U.S. Panel on Cost-Effectiveness in Health and Medicine to create the Childhood Obesity Intervention Cost Effectiveness Study (CHOICES) model.

^c^ Mean and 95% uncertainty intervals reported

#### Policy interventions

Six studies [41–46] estimated the cost and health benefits of city-wide, and state and national policies aimed at preventing future weight gain and obesity (Table 5). Kuo and colleagues [41] developed a simulation model estimating the impact on annual weight gain in Los Angeles (LA) County of a California menu law mandating large restaurant chains to display the caloric content of menu items. The model assumed 10% of all customers would consume 100 calories less per meal and found the law was projected to avert as much as 500,000 lbs. of the estimated annual LA County population weight gain (1.25 million lbs.) in children 5 to 17 years old.

The Northeast Iowa Food and Fitness program enacted multilevel changes during a 6-year long program. The changes targeted schools and home meals and PA, established school gardens, and at the community-level provided access to outdoor recreational spaces and programs, local farmers markets and affordable healthy food. It was shown that children ages 4–12 years who had longer periods of program exposures (2 to 6 years) demonstrated a greater improvement in appropriate growth rate compared with children with shorter periods of program exposure (0 to 1 year) [46].

Dharmasena et al. utilized an economic demand model based on household purchasing habits to assess the impact of a 20% tax on SSB consumption, caloric intake and weight. Results using the most conservative estimate showed an overall reduction in SSB with corresponding increases in fruit juice and low-fat milk consumption. The interrelated changes in beverage consumption patterns was forecast to produce an average reduction of 449 calories per month resulting in a mean body weight reduction of 1.54 lbs/year.

#### Costs and cost effectiveness analysis

Finally, three studies describe the economic impact and health consequences of obesity prevention policies using a Markov-based cohort model to estimate the cost effectiveness of: an excise tax on SSBs, [44] eliminating the tax subsidy for TV advertising, [42] and implementing a set of hypothetical childcare center-based policy changes [45], (Table 5). Sonneville and colleagues [42] found that eliminating the ability of food manufactures to deduct the cost of advertising unhealthy foods would result in a mean BMI reduction 0.028 per child [42]. Likewise, Wright and colleagues [45] estimated that a hypothetical childcare center-based policy (eliminating SSBs, limiting fruit juice, serving low-fat milk, limiting screen time and increasing MVPA) would result in a mean BMI reduction of 0.019 BMI units per child [45]. Finally, Long and colleagues [44] estimated that a tax of $0.01/ounce on SSBs would reduce the total calories consumed by children ages 2–4 and 5–9 years, by -1 to -13 kcal/day, respectively, and result in a mean reduction in BMI of approximately 0.16 units for children 2–19 years. The cost per unit BMI reduction based on these policies ranged from $1.16 for eliminating the advertising subsidy to $57.80 for the childcare center-based policy.

## Discussion

This scoping review identified the characteristics and effectiveness of obesity prevention interventions, programs and policies across the early life course and at multiple levels of influence in the US. There were a number of key findings (S2 Appendix). We found slightly less than half of the interventions initiated during pregnancy, infancy or preschool were effective at improving a weight-based measure of growth or weight status in young children [17, 19, 20, 22–25, 28, 31, 36, 38]. All interventions included strategies to influence health behaviors at an individual or interpersonal level. However, few studies removed obstacles in the physical/built environment, sociocultural environment or healthcare system. The majority of the interventions were conducted in children at higher risk of obesity, in early childcare education settings. The impact of menu labeling laws, taxing SSBs and eliminating incentives for TV advertising of unhealthy foods, on a direct weight-based measure of growth or weight status in children under age 6 years remains unclear. Finally, this review confirmed the lack of available data on the cost of implementing obesity preventions efforts in the US.

We used the NIMHD Research framework [53] to guide our examination of obesity prevention efforts considering factors relevant to obesity and health equality research. We found that all interventions initiated during pregnancy and infancy focused on modifying biological risk factors of obesity by enhancing individual-level knowledge of healthy eating patterns, appropriate gestational weight gain, prolonged breastfeeding, delayed introduction of complementary feeding, responsive feeding techniques and appropriate infant growth patterns [19, 20]. For example, in the primary care setting, MI coaching techniques were applied to reduce obesogenic behaviors [25, 37], while in the childcare center setting, parents and teachers gained the necessary knowledge and skills to serve as role-models of healthy-lifestyle behaviors [19, 28, 36, 38]. Also, preschool children were encouraged to modify their behaviors using culturally adapted, nutrition and PA lessons [19, 28, 31, 36, 38], food group-themed, hand puppet activities [29], English and Spanish-language CDs [30], healthy snacks [22, 28, 36] and structured MVPA [23, 24, 28].

All effective interventions identified in this review incorporated interpersonal-level strategies affecting family behaviors and home routines. Parental and family participation was either a primary or secondary component of all successful interventions. Parents attended culturally tailored education sessions [38], home visits [17, 20, 31], or were assigned homework, received instructional handouts and newsletters promoting frequent family meals, adequate sleep, family PA and limiting screen time [20, 22, 25, 28, 31, 36, 38]. The inclusion of parent-direct strategies in successful interventions is consistent with the findings of a recent review of obesity prevention interventions in early childcare centers. Ward and colleagues [54] found that higher parent engagement in the early childcare settings enhanced the effectiveness of interventions by achieving a positive weight related outcome [54].

Recognizing the influence of physical environments on the risk of childhood obesity and health disparities is critical to the design of multilevel obesity prevention interventions. Yet, less than one-third of the studies aimed to modify a component in the physical/built environment. These interventions effectively removed barriers to facilitate healthier lifestyle behaviors. They included components such as removing TVs from bedrooms, providing alternative playtime activities, and implementing childcare centers polices limiting SSBs, serving water, low-fat milk, fruits and vegetables as snacks; increasing hours of PA, and limiting screen time [22, 31, 36]. At the community-level, Karanja and colleagues [17] enacted tribal-wide policies providing access to breast feeding rooms and reallocating resources by stocking vending machines with water and providing water coolers at community-sponsored activities. Collectively, these types of physical environment changes rendered healthy behaviors as the default behavior.

Approximately half of the interventions integrated strategies to change sociocultural environmental-level factors, with considerable variability in the intensity of the components. Few included primary objectives examining the effectiveness of culturally-tailored training programs [38, 39] or other high-intensity activities such as media campaigns encouraging drinking water and breastfeeding as cultural values [17] or establishing group sessions intended to support shared sociocultural values [19]. While the majority of studies included the following types of moderate- to low-intensity components: using bi-cultural/bilingual interventionalist [19, 25, 29, 30, 32, 36, 38, 39], culturally adapted curriculum [25, 26, 28–31, 33, 34, 36, 38, 39] and providing culturally relevant recipes [19, 25, 26, 35, 39].

Healthcare system-level changes facilitate access to healthcare resources, engage parents in healthcare decisions and improve parent-healthcare provider relationships. Furthermore, the early initiation of interventions in the healthcare setting might alter the course of health and disease, and reduce future health disparities [55]. Yet, of the 5 healthcare-based interventions [16, 19, 21, 25, 37], only two reported improvements in a measure of obesity. One was informed by the chronic care model and used brief MI format [25], while the other used a patient-centered approach and group sessions [19]. In contrast, others failed to improve childhood growth trajectories [16, 21, 37]. Such results suggest that even in a healthcare setting, the intensity of the program and study population are important considerations for the success of any intervention.

This review identified studies that estimated the health and economic impact of regional and national SSBs pricing strategies, labeling laws and food marketing policies. These strategies were shown to change purchasing behavior and improve the prevalence of obesity, and potentially to generate tax revenue, drive the reformulation of unhealthy foods, and change social norms [42, 44]. Although the population reach and societal-level impact of obesity prevention policies are high, the long-term outcome on the prevalence of early childhood obesity remains uncertain.

None of the included interventions reported on the costs or cost effectiveness of the study. The lack of economic evaluations is a surprising finding given that the annual cost of obesity-related medical spending was estimated to exceed $147 billion [56]. Consistent with our findings, Wolfenden and colleagues [57] noted that 88% of the systematic reviews of obesity prevention intervention in children did not report whether a cost analysis has been conducted. In the absence of CEA data, a reliable cost threshold could assist stakeholders to determine the amount of money to spend on obesity prevention interventions. Ma and Frick [48] established the break-even cost of $339 per child as a “good value” for interventions resulting in a 1% reduction in childhood obesity prevalence.

There were several limitations to our review. First, our scoping review presented a comprehensive overview of the quantity and context of current childhood obesity prevention efforts in the US, which limits the generalizability of our findings to other countries. Next, our review did not identify any obesity prevention interventions conducted during pre-pregnancy and identified very few studies conducted during pregnancy. It is likely that our search strategy may not have been sensitive enough to identify the full breadth of research activities during these early life stages. Since we followed standardized methodology for scoping reviews, we did not assess the effect size of the interventions or systematical evaluate the quality of the individual studies including the risk of bias quality. We acknowledge that many of the studies were of varying quality based on study design, sample size, intensity, analytical approaches, high attrition rates and low parent attendance rates. Although we excluded studies judged to be fatally flawed, our results are subject to a range of biases due to these many threats to the internal and external validity of the study results. Furthermore, given that studies reporting positive results are more likely to be published, conclusions may also be subject to publication bias [44]. Finally, our synthesis was based on interventions reporting a weight-based measure of growth or weight status in normal weight children. Because we did not consider favorable behavioral or PA outcomes the generalization of our results may be limited.

## Conclusions

This review presents an overview of the current state of obesity prevention efforts across multiple levels of influence and the early life course in the US. The majority of efforts focused on individual and interpersonal-level health behavior changes in preschoolers. Thus, there is a need to intensify obesity preventive efforts during critical periods of health development and target multiple levels of influence, especially regarding physical, sociocultural and healthcare system-level obesity risk factors. Furthermore, there is considerable uncertainty around estimates of the economic impacts of obesity prevention interventions and policies. Future studies should estimate the feasibility, effectiveness, and cost-effectiveness of obesity prevention interventions and policies. Addressing these research gaps may provide government agencies, policy makers, and healthcare payers with the necessary scientific evidence to make informed decisions regarding the allocation of funds for initiatives aimed at decreasing the prevalence of early childhood obesity.

## Supporting information

S1 Appendix Table of data extraction information for interventions. (DOCX)

S2 Appendix Summary of key findings. (DOCX)

S1 PRISMA PRISMA checklist. (DOC)
