# Correction: Early childhood obesity prevention efforts through a life course health development perspective: A scoping review

**DOI:** 10.1371/journal.pone.0211288

**Published:** 2019-01-17

**Authors:** Sheri Volger, Diane Rigassio Radler, Pamela Rothpletz-Puglia

As a result of the typesetting process, additional gridlines appear in Tables 2–4. The corrected Tables 2–4 are included here as Supporting Information files.

## Supporting information

S1 File Table 2. Characteristics of interventions during pregnancy.
(PDF)

S2 File Table 3. Characteristics of interventions during infancy.
(PDF)

S3 File Table 4. Characteristics of interventions during preschool.
(PDF)
