# Supplementary material for: Early childhood obesity prevention efforts through a life course health development perspective: A scoping review
Source: PLoS One. 2018 Dec 28;13(12):e0209787. doi: 10.1371/journal.pone.0209787 (PMC6310279; doi:10.1371/journal.pone.0209787)
Supplement: S1 Appendix — (DOCX) [file pone.0209787.s001.docx]

Appendix 1. Table of data extraction Information for interventions

| **Data categories** | **Response options** |
| --- | --- |
| Geographic location | Urban, Rural, Suburban, Mixed |
| Study design type | RCT, cRCT, nRCT, Prospective Cohort, Retrospective Cohort, Before and After Study, Other_______ |
| Study population | Pre-pregnancy, Pregnancy, Infancy, Preschool |
| Primary setting | Home, Childcare center, Primary-care provider, Hospital clinic, Community-wide, Health Center, Other______ |
| Primary participants | Child, Parent, Mother, Primary Caregiver, Childcare Center Teacher, Childcare Center Staff, Primary Care Provider, Community Worker, Other, _____ |
| Treatment provider | Parent, Teacher, Pediatrician, Physician, Nurse, clinic Staff, Social worker, Other____ |
| Domains of influence | Biological, Behavioral, Physical/Built Environment, Sociocultural Environment, Healthcare System |
| Levels of Influence | Individual, Interpersonal, Community, Societal |
| Format | Individual Session, Group session |
| Intervention Type | Behavior, Diet, PA, Policy, Cost-effectiveness Analysis |
| Mode of Delivery | Face-to-Face, Email, Phone, Multimedia, Text, Internet, Other________ |
| Obesity Outcomes | Change in: Weight, BMI, BMI-z score, BMI percentile, Other______ |
| The following data categories were collected and recorded with free text: | Name of the program; Location of the study; Study setting; Population inclusions criteria; Sample size, Attrition rate, Effectiveness / statistics |
