# Supplementary material for: Early childhood obesity prevention efforts through a life course health development perspective: A scoping review
Source: PLoS One. 2018 Dec 28;13(12):e0209787. doi: 10.1371/journal.pone.0209787 (PMC6310279; doi:10.1371/journal.pone.0209787)
Supplement: S2 Appendix — (DOCX) [file pone.0209787.s002.docx]

Appendix 2. Summary of key findings

| - Less than half of the interventions improved a measure of obesity in children less than 6 years of age (17, 19, 20, 22-25, 28, 31, 36, 38) |
| --- |
| - All interventions focused on individual or interpersonal levels of behavioral change |
| - Most interventions took place in early childhood education centers (22-24, 26-30, 33-36) |
| - Very few interventions were initiated during pregnancy (16-18) and infancy (19-21) |
| - All effective interventions included parenting and family participation (17, 19, 20, 22-25, 28, 31, 36, 38) |
| - Interventions with MI coaching techniques reported mixed results (25, 31, 37) |
| - Very few interventions focused on the physical or built environment changes (17, 18, 22, 26, 27, 31, 35, 36) and only 4 interventions were effective in improving a measure obesity in young children (17, 22, 31, 36) |
| - The intensity of interventional components aimed at changing sociocultural environmental factors varied from high- to low-intensity. Examples of high-intensity activities included interventions aimed at changing social norms (17) and providing sociocultural support groups (18). While moderate to low intensity activities included: the use of bilingual interventionists (18, 24, 28-30, 35, 37, 38), culturally relevant curriculum (26, 28-31, 33-36, 38, 39) and providing recipes adapted for cultural preferences (19, 35, 36, 38) |
| - Health care system level intervention may be costly (48) and produced inconsistent results (16, 17, 19, 21, 25, 26, 37, 40) |
| - Policy level interventions aimed at labeling laws, (41) local, state and national pricing strategies (43, 44), and food marketing (42) may improve the prevalence of childhood obesity and generate tax revenue |
| - There is a lack of evidence about the cost-effectiveness of pediatric obesity prevention interventions |
