# Supplementary material for: Correction: Early childhood obesity prevention efforts through a life course health development perspective: A scoping review
Source: PLoS One. 2019 Jan 17;14(1):e0211288. doi: 10.1371/journal.pone.0211288 (PMC6336316; doi:10.1371/journal.pone.0211288)
Supplement: S1 File — Characteristics of interventions during pregnancy. (PDF) [file pone.0211288.s001.pdf]

Table 2. Characteristics of interventions during pregnancy

Author<br>Year<br>Study Name	Study location<br>Study Setting	Population	Study Design<br>Analytical Sample [a]	Theoretical Framework	Level of Influence<br>Domains of Influence	Intervention<br>Delivery	Participant<br>Treatment provider	Effectiveness
Gregory [16]<br>2016<br>Nutrition in Pregnancy clinic (NIP)	Baltimore, MD<br>Urban hospital, health care clinic	Pregnant, < 21 weeks gestation; Pre-pregnancy BMI > 30 kg/m2; Medicaid insurance; Mainly AA	Retrospective cohort<br>Mothers: I=61; C=145 Infants: I=32 C=97	Obstetrical model	Individual, interpersonal<br>Biological, Behavioral, Healthcare system	Behavior, Diet, Appropriate GWG<br>Individual sessions	Mother, Infant<br>Physician, Nurse, practitioner, Nutritionist	Not significant: at 1-year, infant WFL ≥ 95th percentile I=17%; C=15%, P = .66
Olson [18]<br>2014<br>Healthy Start Partnership (HSP)	Rural, NY 8-counties with below state median family income; higher rates of childhood overweight/ obesity in 6 of the counties<br>Combination community-wide plus primary care clinic	Pregnant, <24 weeks gestation and 6 months post-partum; infant weights through 6 months	Prospective cohort study<br>Mothers: I=114; C=152; Infants: I=88 C=65	Community coalition action theory	Individual, Interpersonal, Community, Societal<br>Biological: Behavioral, Physical / built, Sociocultural	Environmental community changes, Diet, PA, Appropriate GWG, Breastfeeding<br>Community exposure, multiple formats & modes of delivery	Mother, Infant, Community<br>Multiple sectors	Not significant: at 6 months, WFL z-score in (%), I = 34.2%; C=31.4%, P = .52
Karanja [17]<br>2010<br>The toddler overweight and tooth decay prevention study (TOTS)	Portland Area Indian Health Services (Idaho, Oregon, WA)<br>Combination community-wide plus home-visit/ phone	Pregnant: affiliated with 1 of 3 AI / AN tribes with children at higher risk for overweight	Before and after design<br>Mothers: I=142; C=63; Infants: I=125; C=53	Home-visiting model	Individual, interpersonal, community<br>Biological, Behavioral, Physical/ built, Socio-cultural, Healthcare system	Environmental community changes, Behavior, Diet, Breastfeeding, Reduce SSB<br>Individual, face to face, Phone, Multimedia	Mother, Infant, Community<br>Trained, peer, community worker	**Significant**: at 24 months, BMI- z scores increased less in the community-wide plus home groups (Tribes B & C) compared with community only (Tribe A) (−0.75, P = .016)

Abbreviations: AA, African American; AI, American Indian; AN Alaskan Native; BMI, Body Mass Index kg /m^2, C, comparator group; GWG, gestational weigh gain; I, Intervention group; PA, physical activity; PCP, primary care provider; WFL, weight-for-length; Wt, weight

[a] Sample size is the analytical sample or sample included in the primary analysis
