# Supplementary material for: Correction: Early childhood obesity prevention efforts through a life course health development perspective: A scoping review
Source: PLoS One. 2019 Jan 17;14(1):e0211288. doi: 10.1371/journal.pone.0211288 (PMC6336316; doi:10.1371/journal.pone.0211288)
Supplement: S2 File — Characteristics of interventions during infancy. (PDF) [file pone.0211288.s002.pdf]

Table 3. Characteristics of interventions during infancy

Author<br>Year<br>Study Name	Study location<br>Study Setting	Population	Study Design<br>Analytical Sample [a]	Theoretical Framework	Level of Influence<br>Domains of Influence	Intervention<br>Delivery	Participant<br>Treatment provider	Effectiveness
Machuca [19]<br>2016<br>Well Baby Group (WBG)	Bronx, NY<br>Federally Qualified Healthcare Center	Enrolled in WBG by age 2 months; Attended at least on WBG group and 24- or 30-month well-child care visit	nRCT<br>Mothers: I=47; C=140	Trans-theoretical model stages of change; Social Learning Theory	Interpersonal, Individual, Community<br>Biological, Behavioral, Sociocultural environment, Healthcare system	Behavior and Diet<br>Group mother sessions	Mothers, Infant<br>Pediatrician Registered Dietitian	**Significant**: at 2 years, I group were significantly less likely to have a BMI-for-age $\geq$ 85th percentile compared with C (2.1% vs. 15.0%; OR 0.12; 95% C; 0.02-0.94; P = .02)
Schroeder [21]<br>2015<br>Growing Leaps and Bounds	Baltimore, MD<br>Health Centers	Healthy infants; ≥2000 g birth weight; discharged home < 5 days post birth	Randomized cluster<br>Centers=4; Infants: I=112; C=110	Not stated	Individual, Interpersonal<br>Biological, Behavioral, Healthcare system	Behavior, Diet and PA<br>Individual sessions, Brochures, Phone, Postcards	Parents, Infant<br>Pediatrician; Nurse practitioner; Clinic staff	Not Significant: at age 24 months, no between group difference in growth pattern; for example, mean (SD) BMIz-scores I=[0.339 (1.13) vs.C= 0.218 (0.95), P >.05]
Paul [20]<br>2011<br>Sleeping and Intake Methods Taught to Infants and Mothers Early in life (SLIMTIME)	Hershey, PA<br>Home	Mothers intending to breastfeed; Newborn infants	RCT (2 x 2 design)<br>Mother Infant pairs=110; Soothe/Sleep, n=29; Introduction of solids, n=29; Both, n=22; None, n=30	Not stated	Interpersonal, Individual<br>Biological, Behavioral	Behavior and Diet<br>Individual sessions, face-to-face, video, Instructional handouts	Mothers, Infant<br>Research Nurse	**Significant**: at 1 year, infants in both I groups had lower mean WFL percentiles compared with C (I=33rd percentile vs. C=50th percentile; P = .009)

Abbreviations: BMI, Body Mass Index kg /m2, C, comparator group; I, Intervention group; nRCT, non-randomized control trial; OR, Odds ratio; PA, physical activity; RCT, randomized control trial; SD, Standard deviation; WFL, weight-for-length; WIC, Women, Infants, and Children Program; Wt, weight

[a] Sample size is the analytical sample or sample included in the primary analysis
