# Supplementary material for: Correction: Early childhood obesity prevention efforts through a life course health development perspective: A scoping review
Source: PLoS One. 2019 Jan 17;14(1):e0211288. doi: 10.1371/journal.pone.0211288 (PMC6336316; doi:10.1371/journal.pone.0211288)
Supplement: S3 File — Characteristics of interventions during preschool. (PDF) [file pone.0211288.s003.pdf]

Table 4. Characteristics of interventions during preschool

Author<br>Year<br>Study Name	Study location<br>Study Setting	Population	Study Design<br>Analytical Sample [a]	Theoretical Framework	Level of Influence<br>Domains of Influence	Intervention<br>Delivery	Participant<br>Treatment provider	Effectiveness
Alkon [22]<br>2014<br>Nutrition and Physical Activity Self-Assessment for Child Care	California, Connecticut, North Carolina<br>Licensed child care centers:	3 - 5 years, mainly low income	Cluster RCT (efficacy)<br>Licensed child care centers: I=9; C=8; Children: I=99; C=110	Not Stated	Individual, Interpersonal<br>Biological, Behavioral, Physical/ Built	Behavior, Diet, PA, and Policy<br>Group child sessions; Parent information sheets; Policy changes and consultation sessions	Child care providers; Other staff; Parents<br>Trained nurse; Child care health consultants	**Significant:** at 7 months, significant difference in child-level change in mean BMIz-scores between I and C: Multilevel mode (HLM)I: coeff(SE) -0.14 (0.06); [95% CI; (−0.26, -0.02)]; t statistic (df)(-2.54); P = .02
Annesi [23]<br>2013<br>Start for Life	Southeast USA/ Atlanta, Georgia<br>Childcare center	4 - 5 years; in final year of YMCA affiliated preschool; AA; income at or below US 130% poverty line	Cluster RCT (Efficacy)<br>YMCA-affiliated preschools: I=9; C= 8 Children: I=144; C=129	Social cognitive; Self-efficacy theory	Individual, Interpersonal<br>Biological, Behavioral	Behavior and **PA**<br>Group child exercise, Activity logs, Certificate of accomplishment	Child; Parental support<br>Childcare teacher trained	**Significant**, at 9 months mixed model repeated-measures ANOVA found significant time x treatment effect [F (df 1, 271) = 4.49, P = 0.035) indicating a greater reduction in the change in BMI for the I group
Annesi [24]<br>2013<br>Start for Life	Southeast USA<br>Childcare center	4 - 5 years, in final preschool year; mainly AA (86%); lower to lower-middle class	Cluster RCT (Efficacy)<br>YMCA-affiliated preschools: I=18; C=8 Children: I=690; C=464	Social cognitive; Self-efficacy theory	Individual, Interpersonal<br>Biological, Behavioral	Behavior and **PA**<br>Group child exercise, Activity logs, Certificate of accomplishment	Child; Parental support;<br>Childcare teacher trained	**Significant**: at 9 months mixed model repeated-measures ANOVA found significant time x treatment effect [F (df 1,1152) = 5.16, P = 0.023) indicating a greater reduction in the change in BMI for I group
Fitzgibbon [28]<br>2005	Chicago, Illinois	3 - 5 years, attends HS; low-income; primarily black (99%)	Cluster RCT (Efficacy)	Social cognitive theory; Self-determina-tion theory; Trans theoretical	Individual, Interpersonal	Behavior, Diet and **PA**	Child, Parents	**Significant**: a smaller increase in BMI for I vs. C at 1 year [-0.62 (95% CI -0.95, -028), P = .002] and 2 years [-0.65 (95% CI; -1.09 to -0.21) P = .008]

Hip-Hop to Health Jr.	Childcare center		HS Centers: I=6; C=6 Children: I=143; C=146	model- stages of change -	Biological, Behavioral, Sociocultural	Group child sessions; Newsletter, Homework	Trained childhood educators (research team member)	
Natale [36]<br><br>2017<br><br>Healthy Caregivers-Healthy Children	Miami-Dade County, Florida<br><br>Childcare center	2 - 5 years; low-income children; racial/ ethnic distribution of Miami-Dade County: 60% Hispanic, 20% AA	Cluster RCT (Effectiveness)<br><br>Subsidized Child care centers: I=12: C=16: Children: I=754; C=457	Socio-ecological model; Social Cognitive Theory	Individual, Interpersonal<br><br>Biological, Behavioral, Physical/ Built, Sociocultural	Behavior, Diet, PA and Policy<br><br>Policy changes; Group teacher, parent and child sessions; English and Spanish resources; Newsletters, Homework	Child care providers and staff; Parents, Child<br><br>Childcare teacher trained; Bilingual study team members	**Significant:** at 2 years, a growth curve analysis showed a significantly smaller increase in PBMI for I vs. C [negative slope (ß coefficient= -1.95, SE = 0.97, P = .04)
Lumeng [34]<br><br>2017<br><br>Preschool Obesity Prevention Series [POPS]	Urban and Rural Michigan<br><br>Childcare center	Child attending HS, first year	Cluster RCT (Efficacy)<br><br>HS classes: I=9; C=9; I Obesity-prevention, n=221; I2= plus self -regulation, n=253); C=216	Social cognitive theory; observational learning/ reinforcement techniques	Individual, Interpersonal,<br><br>Biological, Behavioral, Sociocultural	Behavior and Diet<br><br>Group child and parent sessions, Video vignettes, Homework, Phone calls	Child; Parents<br><br>Master's-level nutrition/ mental health specialist; Childcare teacher trained	Not significant: at the end of the academic year, no between group difference in the prevalence of overweight or obesity and BMI-z scores (All, P >.05)
Kong [33]<br><br>2016<br><br>Hip-Hop to Health Jr.	Chicago, Illinois<br><br>Childcare center	3 -5 years, HS serving AA, low-income families	Cluster RCT (Effectiveness)<br><br>HS centers: N=18; Children; I=285; C=258	Social cognitive theory; Self-determination theory; Trans-theoretical model - stages of change	Individual, Interpersonal<br><br>Biological, Behavioral, Sociocultural	Behavior, Diet and **PA**<br><br>Group child sessions: Exercise CD, Newsletter, Homework	Child, Parents<br><br>Childcare teacher trained	Not significant: at 1 year, no between group difference in adjusted mean changes in BMIz scores (P = .83)
Esquivel [27]<br><br>2016	Oahu, Hawaii	HS classroom; 2 to 5 years; NHPI children (23%)	Cluster RCT (Effectiveness)	Not stated	Individual, Interpersonal,	Behavior, Diet, PA and Policy	HS Teachers; Child	Not significant: at 7 months, no within - group differences in mean change in BMIz-scores and BMI categories (All, P >.05)

Children's Healthy Living Program (CHL)	Childcare center		HS classes (geographical cluster): I=11; C=12; Children: I=114; C=132		Biological, Behavioral, Physical/ Built	Group teacher sessions, Menu changes, Classroom nutrition / PA resources, Newsletters, Phone	Childcare teacher trained	
Natale [35]<br>2014<br><br>Healthy Inside–Healthy Outside	Miami-Dade County, Florida<br><br>Childcare center	2 to 5 years, child care centers serving multi-ethnic children from low-income families	Cluster RCT (Effectiveness)<br><br>Subsidized child care centers: I=6; C=2; Children: I=238; C= 69	Socio-ecological model	Individual, Interpersonal<br><br>Biological, Behavioral, Physical/ Built, Sociocultural	Behavior, Diet, **PA** and Policy<br><br>Policy changes; Group sessions, Spanish and English resources; Newsletters, Homework	Child care providers; Other staff; Parents; Child<br><br>Childcare teacher trained; RD/ Nutritionist	Not significant: at 12 months no between group difference in mean Wt. (P = .35) and BMI-z scores (P = .81)
Fitzgibbon [30]<br>2013<br><br>Family-based Hip-Hop to Health	Chicago, Illinois<br><br>Childcare center	3 - 5 years, low-income, Latino	Cluster RCT (Feasibility)<br><br>HS centers: I=2, C=2: Children: I=61; C=67	Social cognitive theory; Health belief model; Self-Determination Theory	Individual, Interpersonal<br><br>Biological, Behavioral, Sociocultural	Behavior, Diet and **PA**<br><br>Group child sessions, Nutrition and Spanish exercise CD, Parent group sessions and PA, Newsletters	Child, Parents<br><br>Trained, bilingual/ bicultural educator	Not significant: at 1 year, did find a greater reduction in in BMI and BMIz-scores in the I group (P >.05)
Fitzgibbon [29]<br>2006<br>Hip-Hop to Health Jr. Latino	Chicago, Illinois<br><br>Childcare center	3-5 years, low income; mainly Latino HS centers	Cluster RCT (Efficacy)<br><br>HS centers: I=6, C=6; Children: I=176; C=160	Social cognitive theory; Self-determination theory	Individual, Interpersonal<br><br>Biological, Behavioral, Sociocultural	Behavior, Diet and **PA**<br><br>Group child sessions; Lessons in English and Spanish: Spanish exercise CD; Newsletter, Homework	Child, Parents<br><br>Trained, bilingual/ bicultural early childhood educator (research team member)	Not significant: at 1 and 2 years, no between group differences in change in BMI and BMIz-scores (P = .05)

Haines [32]<br>2016<br>Parents and Tots Together	Boston, MA<br>Community health center	2 -5 years; Hispanic (58%) and Black/AA (23%) recruited from community resources serving low-income families	RCT (Efficacy)<br>Parent-child dyad: I=46, C=50	Social contextual framework	Individual, Interpersonal<br>Biological, Behavioral	Behavior, Diet and **PA**<br>Group child and parent sessions, DVD set, Newsletter, Homework, (bilingual interviews)	Parents, Child<br>Trained facilitator	Not significant: at 9 months, no between group difference in BMI (P = .41)
Slusser [38]<br>2012<br>Pediatric Overweight Prevention through Parent Training Program	Los Angeles, CA<br>Community center/ health centers	Parent of 2 - 4 years; Low-income Latino	RCT (Pilot)<br>Local centers: Parent-child dyad: I=44; C=37	Social learning framework	Individual, Interpersonal<br>Biological, Behavioral, Sociocultural	Behavior, Diet and PA<br>Group, Spanish, parent training sessions; Spanish handouts; Homework	Mother, Child (Wt. only)<br>Trained, bilingual staff; social worker or master's level health educator	**Significant:** at 1 year, decrease in mean (SD) PBMI in the I group [-3.85 (0.29) vs. an increase in C group [+1.33 (0.30)]; accounting for drop-out rates with multiple imputation, significant difference shown between change in BMI-z scores [0.24 (0.01) P<.04]
Cloutier [25]<br>2015<br>Steps to Growing Up Healthy	Hartford, CT<br>Pediatric Primary Care Clinic	Caregiver of 2 - 4 years; Hispanic (82%) / AA WIC recipient	N-RCT (Efficacy)<br>Clinics, N=32; Parent-child dyad: I=200, C=218	Chronic care model	Individual, Interpersonal<br>Biological, Behavioral, Sociocultural Healthcare System	Behavior, Diet and PA<br>Individual MI sessions, English and Spanish resources; Handouts, Self-monitoring calendar, Toolkit	Mother, Child (Wt only)<br>Trained primary care clinicians and nurses; bilingual team members	**Significant:** at 12 Months, significant intervention effect on change in PBMI (ß coefficient= -0.23; 95% CI; -0.33, -0.13) with a mean decrease in I (-0.33) compared with an mean increase PBMI (8.75) in the C group (P<.001)
Sherwood [37]<br>2015<br>Healthy Homes/ Healthy Kids-Preschool	Minneapolis-St Paul area<br>Pediatric Primary Care Clinics/ Phone	Families with a 2- to 4-year-old with a scheduled well-child visit; BMI or weight-for-height age and sex percentile from 50th to 95th; one overweight parent	RCT (Pilot)<br>Parent-child dyad I=30, C=30	Social ecological models; Social cognitive theory	Individual, Interpersonal<br>Biological, Behavioral, Healthcare system	Behavior, Diet and PA<br>Individual MI session, Flipchart, Handouts: Phone MI sessions	Parent; Child (Wt. only)<br>Pediatric PCP counseling; clinic staff; Trained coaches	Not Significant: at 6 months no difference in PBMI (P = 0.64) and BMI z-scores (P = 0.89); post hoc analysis of baseline child weight status moderated the time by treatment effect on BMI percentile (P = .04)

Woo Baidal [40]<br>2017<br><br>MA-CORD WIC	Fitchburg and New Bedford, MA<br><br>WIC Sites	2 - 4 years, WIC participant	N-RCT (Efficacy)<br><br>WIC centers I=2, C=1 Children: I site1=198; I site2=637; C=626	Chronic care model; Energy gap model; Social cognitive theory	Individual, Interpersonal, Community<br><br>Biological, Behavioral Healthcare system	Behavior, Diet and PA<br><br>Train-the-trainer group sessions, Individual parent sessions, Handouts; Healthy weight clinic referrals	WIC - Providers; Parents Child (Wt. only)<br><br>Trained WIC providers	Not Significant: Over 2 years, no significant difference in BMIz-scores adjusting for age, gender, race, ethnicity (P>0.05); Sensitivity analysis, excluding Asian children found site I2 had a significant decrease in BMI-z scores [-0.08 units/year (95% CI, -0.14, -0.02), P = 0.01] compare with the C group
Davis [26]<br>2016<br><br>Child Health Initiative for Lifelong Eating and Exercise (CHILE)	Albuquerque NM<br><br>Childcare center plus local community component	Under 4-years followed for 1 to 2 years; HS serving rural, Hispanic and AI, low-income families	Cluster RCT (Efficacy)<br><br>HS centers: N=16; Children I=945, C=871	Social ecological model	Individual, Interpersonal, Community<br><br>Biological, Behavioral Physical/ built, Sociocultural, Healthcare system	Behavior, Diet, **PA** and Policy<br><br>Group child sessions, English and Spanish resources; Teacher and foodservice training; Family events; Grocery store component, Healthcare provider support	Child; Parents; Family; HS teachers and food service<br><br>Childcare teacher trained; Grocery store; Healthcare provider	Not significant: at 6 months, no between group difference in change in mean BMIz-scores (P = .69) and 2 years (P >.30)
Haines [31]<br>2013<br><br>Healthy Habits, Happy Homes	Boston, MA<br><br>Home/ phone	2 – 5 years; low-income; racial / ethnic minority; television in the child's bedroom	RCT (Effectiveness)<br><br>Parent-child dyad: I =55; C=56	Not stated-applied (MI coaching)	Individual, Interpersonal<br><br>Biological, Behavioral, Physical/ built, Sociocultural	Behavior and Diet<br><br>Individual, home MI sessions, Phone, Mail, Text messages	Families, Child<br><br>Trained, bilingual, health educators	**Significant**: at 6 months, mean BMI decreased in I group (-0.18) but increased in C group (+0.21) with a difference of −0.40 (95% CI, −0.79 to 0.00; P = .05).

Sun [39]<br>2017<br>No study name provided	San Francisco Bay Area, CA<br>Internet-based	Child: 3 – 5 years; attends HS; Low income, Chinese mothers speak / read Cantonese with a BMI ≥ 23 or waist circum. >31.5	RCT (Pilot)<br>Parent-child dyad: I-=16; C=16	Information Motivation behavior model	Individual, Interpersonal<br>Biological, Behavioral, Sociocultural	Behavior, Diet and PA<br>Online/tablets computer: Interactive, Cantonese, modules, Animated short videos, Talk show format	Mothers, Child (Wt. only)<br>Lessons developed by bilingual/ bicultural RDs and health educators	Not Significant: at 6 months, no difference in post-baseline assessment in child's BMI (t=1.21, P=0.24)

Abbreviations: AA, African American; AI, American Indian; BMI, Body Mass Index kg /m^2, C, comparator group; CI confidence interval; coeff(SE), coefficient estimate (standard error); circum, circumference; f, f-test statistic; GWG, gestational weigh gain; HLM, Hierarchical linear modeling; HS, Head Start; I, Intervention group; MI, Motivational Interview; NHPI, Native Hawaiian and Pacific Islander; PA, physical activity; PBMI, BMI percentile; PCP, primary care provider; RCT, randomized control trial; RD, registered dietitian; SD, Standard deviation; t, t-test statistic; vs, versus; WFL, weight-for-length; WIC, Women, Infants, and Children Program; Wt, weight

[a] Sample size is the analytical sample or sample included in the primary analysis

Bold PA- Direct provision of structured PA
